# Supplementary material for: MicroRNA-21 promotes pancreatic β cell function through modulating glucose uptake
Source: Nat Commun. 2022 Jun 21;13:3545. doi: 10.1038/s41467-022-31317-0 (PMC9213410; doi:10.1038/s41467-022-31317-0)

Supplementary Information

MicroRNA-21 Promotes Pancreatic β cell Function through Modulating Glucose Uptake

Ruiling Liu[1, 2 #], Cuilian Liu[3, #], Xiaozhen He[2], Peng Sun[4], Bin Zhang[2], Haoran Yang[3], Weiyun Shi[2 *], Qingguo Ruan[2, 3 *]

1 School of Basic Medicine, Qingdao University, Qingdao, People's Republic of China, 266071
2 State Key Laboratory Cultivation Base, Shandong Provincial Key Laboratory of Ophthalmology, Shandong Eye Institute, Shandong First Medical University & Shandong Academy of Medical Sciences, Qingdao, People's Republic of China, 266071
3 Center for Protein and Cell-Based Drugs, Institute of Biomedicine and Biotechnology, Shenzhen Institutes of Advanced Technology, Chinese Academy of Sciences, Shenzhen, People's Republic of China, 518055
4 Department of Hepatobiliary and Pancreatic Surgery, The Affiliated Hospital of Qingdao University, Qingdao, People's Republic of China, 266000

[#]These authors contributed equally to this work.
***Corresponding authors:** Qingguo Ruan, Ph.D., E-mail address: ruanqg222@hotmail.com; Weiyun Shi, M.D., E-mail address: weiyunshi@163.com

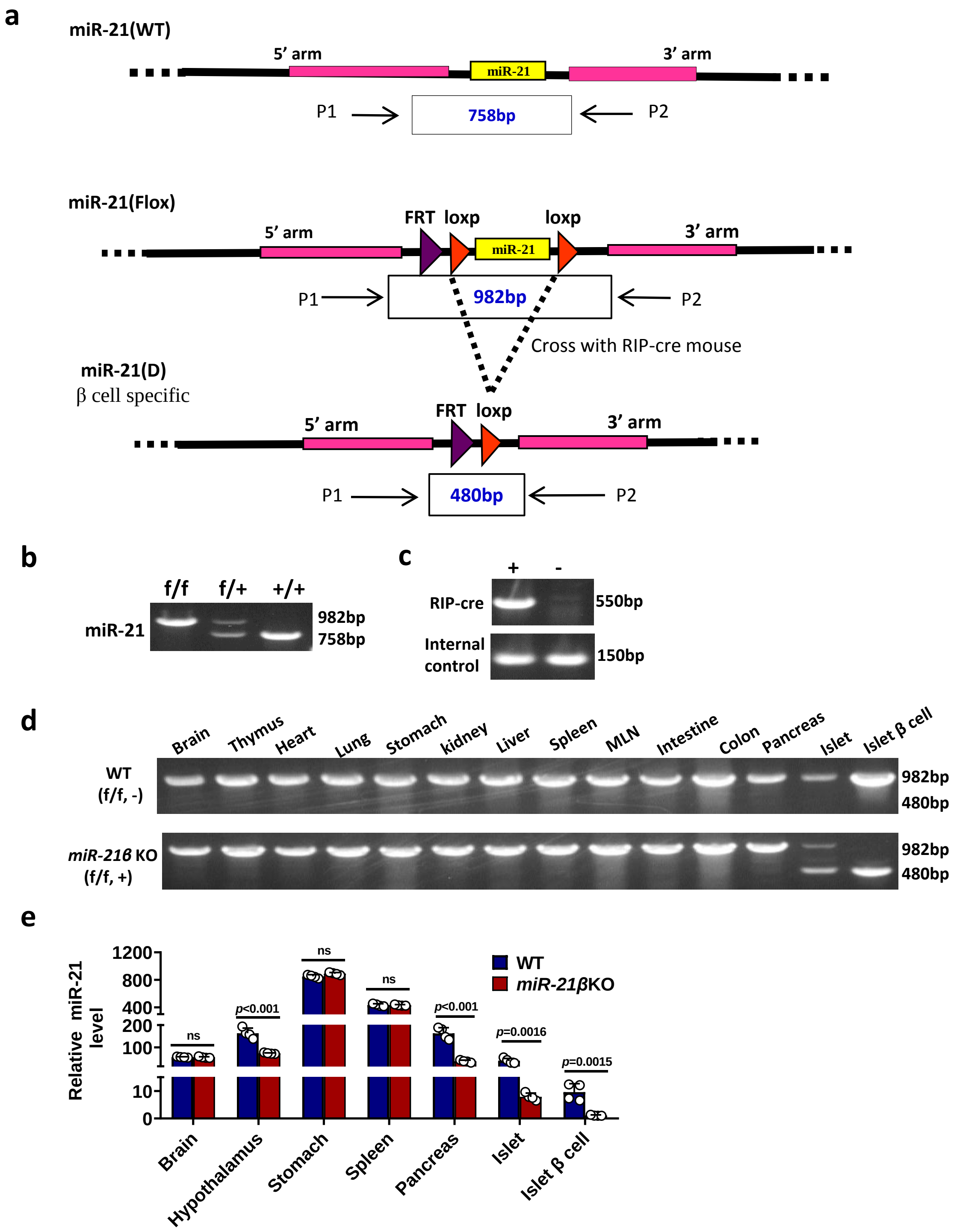

Figure S1. Generation and characterization of *miR-21β*KO mice. (a) Strategy used to generate miR-21 mutant mice by homologous recombination. miR-21 sequence was flanked with loxP sites and recombination induced by breeding mice with Ins2-Cre transgenics. **(b)** miR-21 genotyping was performed with tail genomic DNA from wild type (+/+), heterozygote (f/+) and homozygote (f/f) mice. **(c)** Ins2-Cre genotyping was performed with tail genomic DNA from Cre positive (+) and negative (-) mice. **(d)** miR-21 genotyping was performed with tail genomic DNA from homozygote (f/f) mutant mice crossed with (+) or without (-) Ins2-Cre transgenics. **(e)** miR-21 level in multiple tissues as indicated in the figure was determined by RT-PCR. Data are presented as means ± SD for n=3 biologically independent samples. Statistical significance was analyzed using two-sided unpaired *t*-test and *P* values are indicated in the figure. ns: not significant. All results are representative of at least two independent experiments. Source data are provided as a Source Data file.

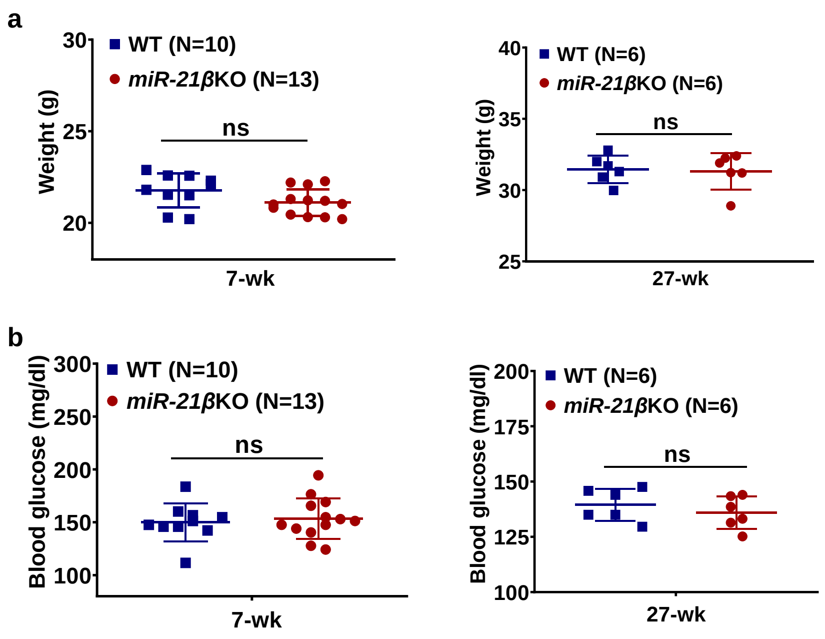

Figure. S2. WT and *miR-21β*KO mice exhibit no significant difference in body weight and blood glucose level. WT and *miR-21β*KO mice were fed with regular diet for 7 or 27 weeks, and body weight **(a)** and 5 h fasting blood glucose level **(b)** were measured. Data are presented as means $\pm$ SD for multiple biologically independent mice (as indicated in the figure). Statistical significance was analyzed using two-sided unpaired *t*-test. ns: not significant. Results were combined from two independent experiments. Equal numbers of male and female mice were used in two genotypes. Source data are provided as a Source Data file.

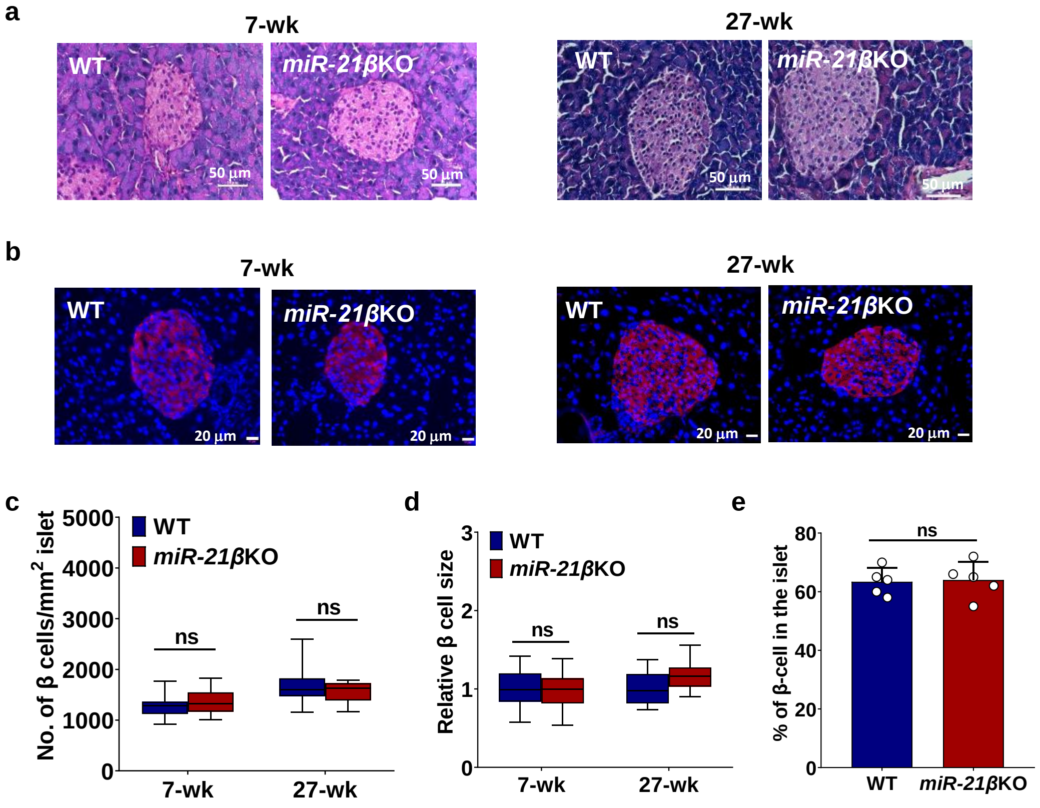

Figure S3. The number, size, and percentage of islet β cells are comparable between WT and *miR-21β*KO mice. WT and *miR-21β*KO mice were fed with regular diet for 7 or 27 weeks. **(a)** Representative images of pancreatic tissue sections stained with hematoxylin and eosin. Scale bar: 50μm. **(b)** Representative images of pancreatic tissue sections stained with primary antibody against insulin (red) and secondary antibody against Alexa Fluor 594-conjugated Donkey anti-Rabbit IgG (H+L). DAPI was used to stain the nuclear (blue). Scale bar: 20μm. **(c & d)** Pancreatic tissues were treated as in (b) and number of β cells/mm^2 islet area was determined by examining insulin positive cells **(c)**. Relative islet β cell size was determined by examining insulin-stained area divided by the number of DAPI-positive nuclei **(d)**. For mice fed with regular diet for 7 weeks, data shown are results combined from 25 islets. For mice fed with regular diet for 27 weeks, data shown are results combined from 16 islets (WT) or 12 islets (*miR-21β*KO) and presented using box and whisker plot. The line within the box represents the median value. The bottom line of the box represents the 1st quartile. The top line of the box represents the 3rd quartile. The whiskers extend from the ends of the box to the minimum value and maximum value. **(e)** Pancreatic islets from 8~10-week old WT and *miR-21β*KO mice (n=5) were dissociated into single cells using trypsin digestion and the percentage of islet β cells was determined by examining auto-fluorescent β cells using flow cytometry. Data are presented as means ± SD for n=5 biologically independent samples. All statistical significance was analyzed using two-sided unpaired *t*-test. ns: not significant. All results are representative of two independent experiments. Equal numbers of male and female mice were used in two genotypes. Source data are provided as a Source Data file.

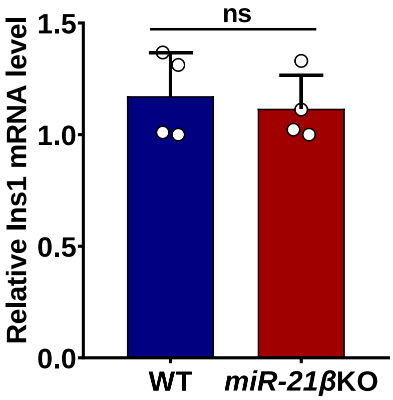

Figure S4. Insulin biosynthesis is comparable between WT and *miR-21β*KO mice. Pancreatic islets were isolated from 7~8-week old WT and *miR-21β*KO (n=4) mice. Total RNA was extracted and relative mRNA expression level of insulin1 was determined by quantitative RT-PCR. Data are presented as means ± SD for n=4 biologically independent samples. Statistical significance was analyzed using two-sided unpaired *t*-test. ns: not significant. Results are representative of two independent experiments. Equal numbers of male and female mice were used in two genotypes. Source data are provided as a Source Data file.

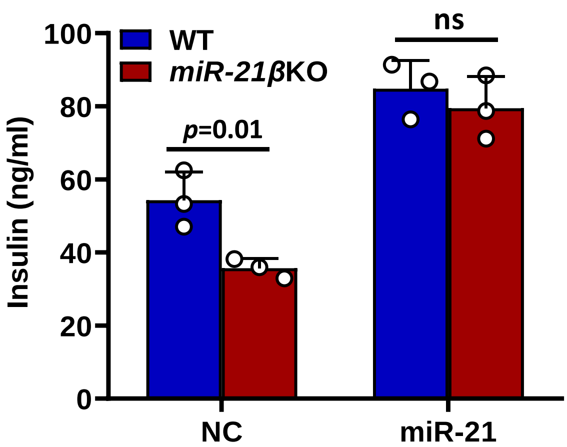

Figure S5. Over-expression of miR-21 rescues the impaired glucose-stimulated insulin secretion by islets from *miR-21β*KO mice. Pancreatic islets from 7~8-week old WT and *miR-21β*KO mice (n=3) were infected with negative control virus (NC) or adenovirus over-expressing miR-21 (miR-21). Virus infected islets were treated with 16.7 mM glucose and insulin level in the supernatant was measured. Data are presented as means ± SD for n=3 biologically independent samples. Statistical significance was analyzed using two-sided unpaired *t*-test and *P* value is indicated in the figure. ns: not significant. Results are representative of two independent experiments. Equal numbers of male and female mice were used in two genotypes. Source data are provided as a Source Data file.

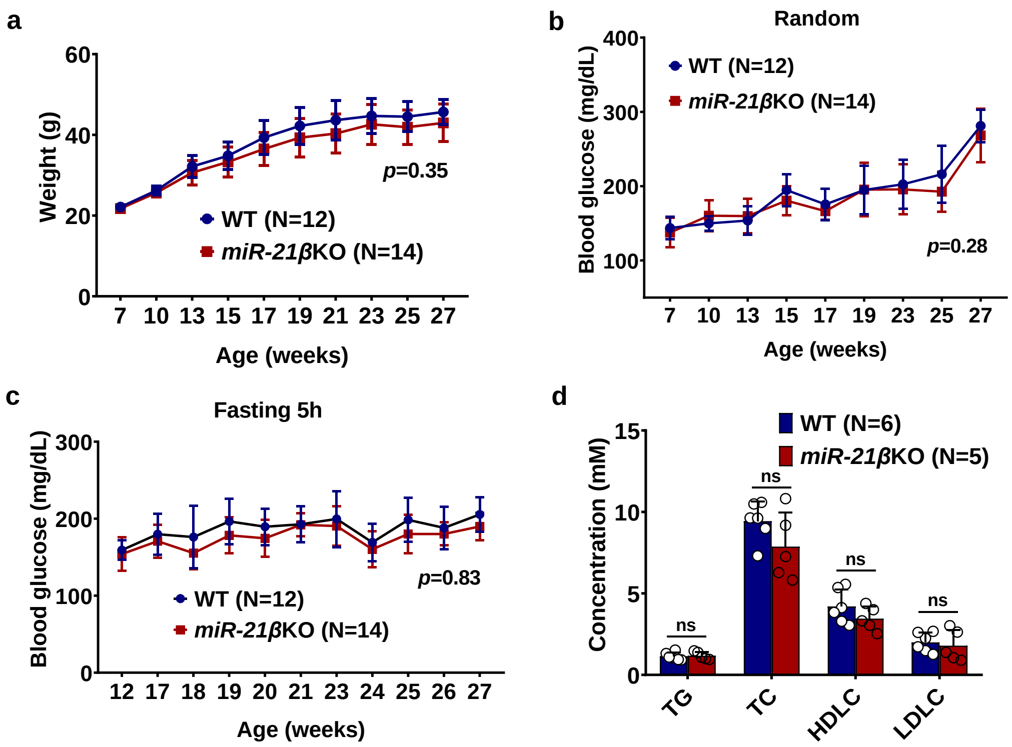

Figure S6. WT and *miR-21β*KO mice fed with high-fat diet exhibit no significant difference in body weight, blood glucose level and lipid/cholesterol metabolism. WT and *miR-21β*KO mice were fed with high-fat diet start from 7~8-week old. The body weight **(a)**, random blood glucose level **(b)** and the 5 h fasting blood glucose level **(c)** were measured at the indicated time-points. For (a-c), data were combined from two independent experiments and presented as means ± SD for multiple biologically independent mice (as indicated in the figure). Statistical significance was analyzed using two-way ANOVA and *P* values are indicated in the figure. *P* values for all comparisons were not significant. At the end of the experiment (week 27), the serum levels of TG, TC, HDLC and LDLC were measured using commercially available kit **(d)**. For (d), data are presented as means ± SD for multiple biologically independent samples (as indicated in the figure). Statistical significance was analyzed using two-sided unpaired *t*-test. ns: not significant. Results are representative of two independent experiments. TG: triglycerides; TC: total cholesterol; HDLC: high density lipoprotein cholesterol; LDLC: low density lipoprotein cholesterol. Equal numbers of male and female mice were used in two genotypes. Source data are provided as a Source Data file.

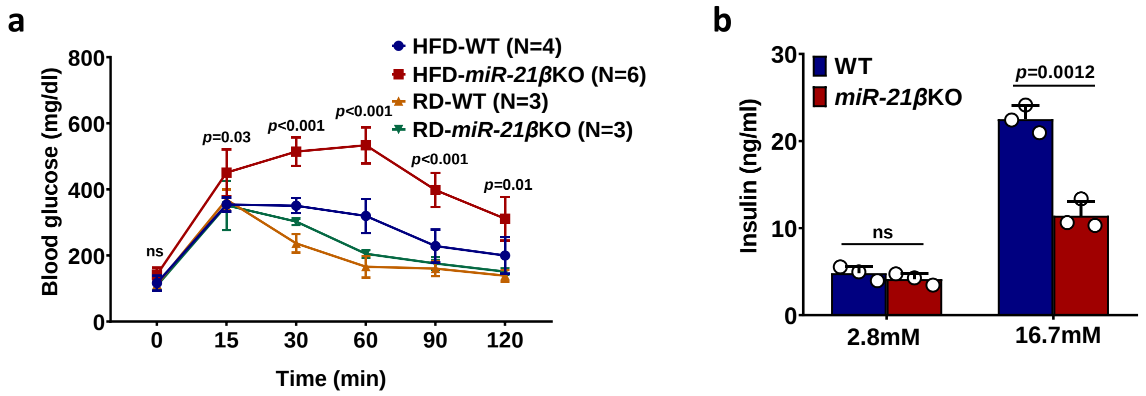

Figure S7. Blood glucose intolerance was aggravated in *miR-21β*KO mice fed with high-fat diet. WT and *miR-21β*KO mice were fed with high-fat diet (HFD) start from 7-8-week old. At the end of the experiment (week 27), mice were fasted for 16 h before treated with 1g/kg glucose, and blood glucose levels were measured at the indicated time-points. As a control, WT and *miR-21β*KO mice fed with regular diet (RD) were also treated the same way as mice fed with high-fat diet, and blood glucose levels were measured at the indicated time-points **(a)**. Data are presented as means ± SD for multiple biologically independent mice (as indicated in the figure). Statistical significance of the difference between WT and *miR-21β*KO mice fed with high-fat diet was analyzed using two-way ANOVA and *P* values are indicated in the figure. Additionally, islets were isolated from WT and *miR-21β*KO mice fed on high-fat diet and treated with 2.8 mM or 16.7 mM glucose. Insulin secretion in the supernatant was measured **(b)**. Data are presented as means ± SD for n=3 biologically independent samples. Statistical significance was analyzed using two-sided unpaired t-test and *P* value is indicated in the figure. ns: not significant. Results shown are representative of two independent experiments. Equal numbers of male and female mice were used in two genotypes. Source data are provided as a Source Data file.

Supplemental Table 1. **Identification of putative miR-21 binding site using TargetScan.**

Functional class	Gene name	miR-21a-5p binding site	miR-21a-3p binding site
**Potassium ion transport**	Abcc9	N	N
	Kcna2	N	N
	Hcn1	N	N
	Actn2	N	N
	**Ptger3**	Y(3315-3321)	N
	**Pten**	Y(434-451)	N
	Adrb2	N	N
	Slc9a9	N	N
	Kcnk10	N	N
	Lrrc38	N	N
	Kcnk9	N	N
	Kcnj13	N	N
	**Scn4a**	Y(1810-1816)	N
	Kcnf1	N	N
	Kcnj2	N	N
	**Lrrc55**	Y(968-974,2337-2344)	N
	**Kcnj5**	Y(1143-1149)	N
**Negative regulation of glucose transmembrane transpor**	Fabp5	N	N
	Pid1	N	N
	Myc	N	N
	Esr1	N	N
**Negative regulation of secretion**	Serpinb1a	N	N
	Entpd1	N	N
	Tnfrsf1a	N	N
	**Adtrp**	Y(516-523)	N
	**Egf**	Y(255-261)	N
	**Ptger3**	Y(3315-3321)	N
	Ezr	N	N
	**Acvr1c**	Y(286-293)	N
	Cd84	N	N
	Acsl4	N	N
	Pde8b	N	N
	Nucb2	N	N
**Negative regulation of transmembrane transport**	Pid1	N	N
	Fabp5	N	N
	Actn2	N	N
	Esr1	N	N
	Myc	N	N
	Gsto1	N	N
	**Ptger3**	Y(3315-3321)	N
	**Pten**	Y(434-451)	N

Y: binding site found; **N:** binding site not found.

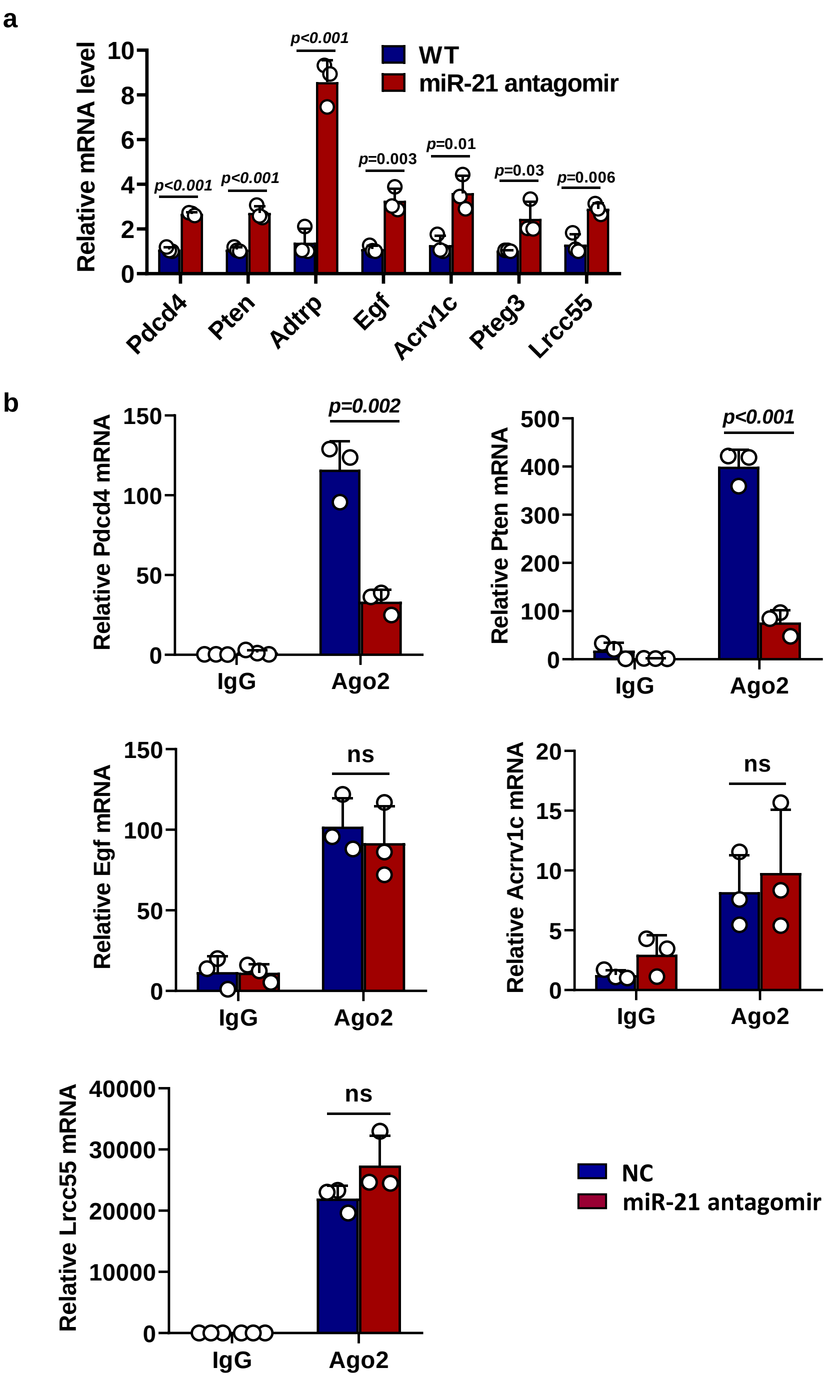

Figure S8. Validation of functional target of miR-21 using Ago2 immunoprecipitation. MIN6 cells were transfected with control (NC) or miR-21 antagomir. **(a)** The expression of potential miR-21 target was examined by RT-PCR. **(b)** Normalized RT-PCR results for enriched mRNA level of potential miR-21 targets in Ago2 immunoprecipitated total RNA. Data are presented as means ± SD for n=3 biologically independent samples. Statistical significance was analyzed using two-sided unpaired *t*-test and *P* values are indicated in the figures. ns: not significant. Results are representative of two independent experiments. Source data are provided as a Source Data file.

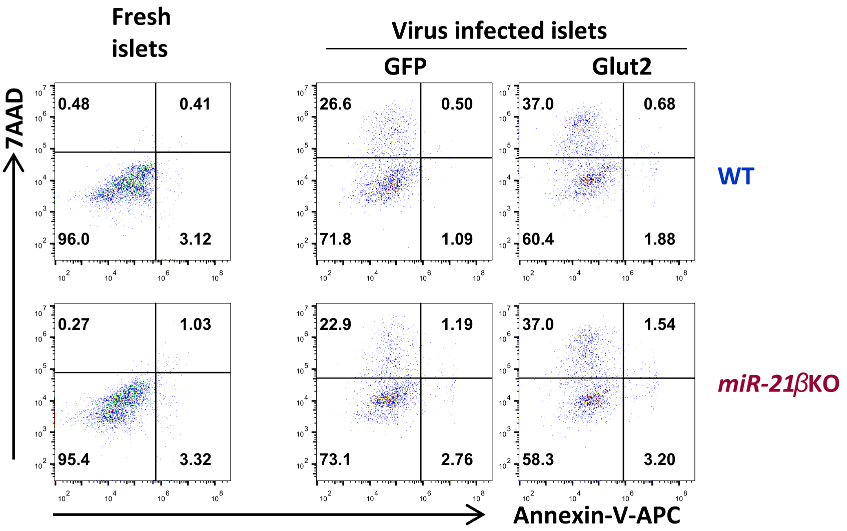

Figure S9. Islets from WT and *miR-21β*KO mice exhibit no significant difference in apoptosis after adenovirally mediated Glut2-overexpression. Pancreatic islets from WT or *miR-21β*KO mice (n=3) were infected with adenovirus expressing negative control (GFP) or murine Glut2 (Glut2). After 24 h, islets were first dissociated into single cells using trypsin digestion and then stained with Annexin-V-APC and 7AAD, and analyzed by flow cytometry. Islets not infected with adenovirus were used as control. Results are representative of two independent experiments. Equal numbers of male and female mice were used in two genotypes. Source data are provided as a Source Data file.

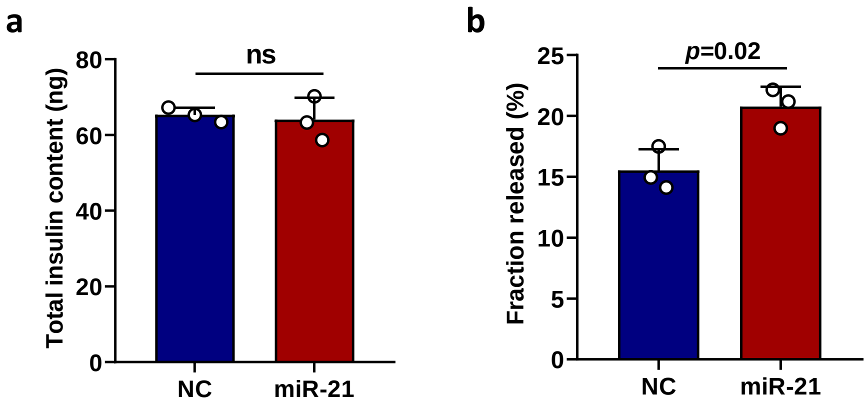

Figure S10. Adenovirally mediated elevation of miR-21 in mouse islets significantly increased fractional insulin release. Pancreatic islets were isolated from 7~8-week old C57BL/6 male mice and infected with negative control virus (NC) or adenovirus over-expressing miR-21 (miR-21). Virus infected islets were treated with 16.7 mM glucose and total islet insulin content **(a)** and fractional insulin release **(b)** were determined by ELISA. Data are presented as means ± SD for n=3 biologically independent samples. Statistical significance was analyzed using two-sided unpaired *t*-test and *P* value is indicated in the figure. ns: not significant. Results shown are representative of two independent experiments. Source data are provided as a Source Data file.

Gating strategies

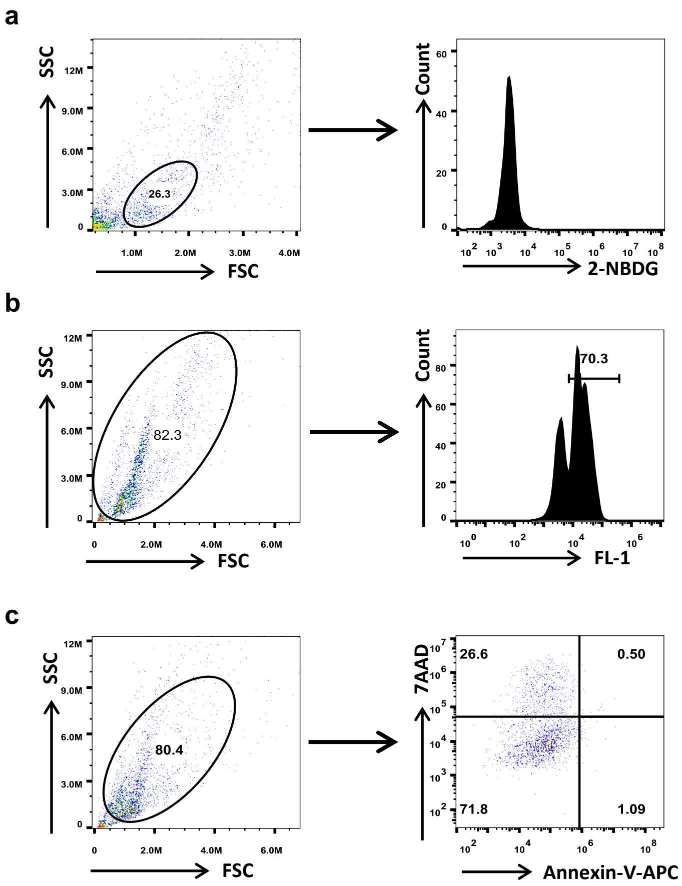

a. Gating strategy for Figure 4d. For glucose uptake measurement, gating is based on the size of pancreatic β cell and the selection of live cells.
b. Gating strategy for Figure S3e. Live cells were gated for the determination of percentage of β-cell in the islet.
c. Gating strategy for Figure S9. Apoptosis analysis was performed on cell population from which debris was gated out.

Uncropped scans of gels in Supplementary Figures

Figure S1B & S1C:

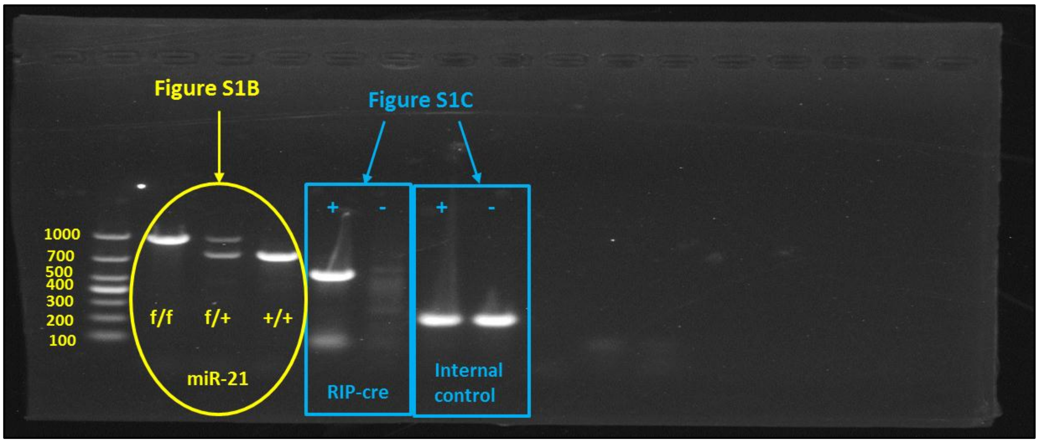

Figure S1D:

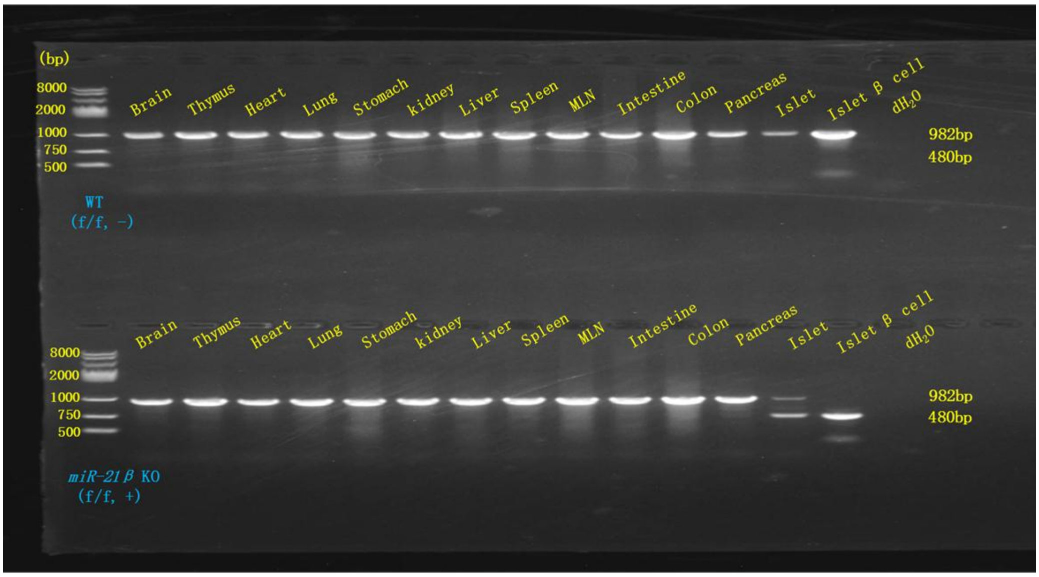

Supplement: Supplementary file 1 — Supplementary Information [file 41467_2022_31317_MOESM1_ESM.pdf]
